# The Role of Human Endogenous Retroviruses in the Initiation and Progression of Melanoma

**DOI:** 10.3390/biomedicines13071662

**Published:** 2025-07-08

**Authors:** Yao Lin, Rosanna Rita Satta, Elena Rita Simula, Shijie Tang, Paola Molicotti, Antonio Cossu, Corrado Rubino, Leonardo Antonio Sechi

**Affiliations:** 1 Department of Biomedical Sciences, University of Sassari, 07100 Sassari, Italy; linyao1163@163.com (Y.L.); ersimula@uniss.it (E.R.S.); molicott@uniss.it (P.M.); 2 Department of Plastic Surgery and Burn Center, Second Affiliated Hospital, Shantou University Medical College, Shantou 515051, China; sjtang3@stu.edu.cn; 3 Department of Medicine, Surgery and Pharmacy, University of Sassari, 07100 Sassari, Italy; rrsatta@uniss.it (R.R.S.); cossu@uniss.it (A.C.); corubino@uniss.it (C.R.); 4 Plastic Surgery Unit, University Hospital Trust of Sassari, 07100 Sassari, Italy; 5 Complex Structure of Microbiology and Virology, Azienda Ospedaliera Universitaria Sassari, 07100 Sassari, Italy

**Keywords:** human endogenous retrovirus, melanoma, epigenetic dysregulation, immune response, treatment

## Abstract

Human endogenous retroviruses (HERVs), as remnants of ancient exogenous retroviruses in the human genome, have received increased attention regarding their pathogenic effects caused by abnormal activation. In normal somatic cells, HERVs are tightly regulated by epigenetic mechanisms and are rarely expressed. In cancer cells, likely due to epigenetic dysregulation, HERVs become abnormally activated and are transcribed and expressed. The innate and adaptive immune responses triggered by HERV activation are closely associated with cancer initiation and progression. Melanoma, as a malignant tumor, often exhibits a poor prognosis in advanced-stage patients. HERVs have been found to be expressed in melanoma and linked to its malignant transformation. Here, we review the potential roles HERVs may play in melanoma development. As promising therapeutic targets for melanoma, research on HERVs could facilitate the development of novel treatment strategies.

## 1. Introduction

Melanoma is a malignant tumor that originates from melanocytes and has the highest mortality rate among skin cancers [1,2]. Melanocytes are pigment-producing cells derived from the neural crest [3]. Over the past few decades, the incidence of melanoma has been on the rise, especially in fair-skinned populations and elderly populations [4]. Melanomas confined to the original site are most likely curable with surgical resection. Once melanoma has metastasized locally or distantly, patient survival is greatly shortened. Researchers have conducted extensive exploration on the initiation and progression of melanoma, from external environmental influences to internal gene mutations. Immunotherapy, targeted therapy, and other new therapeutic approaches have been developed and applied to patients with melanoma [5].

Human endogenous retroviruses (HERVs) are viral sequences integrated into the human genome that result from multiple infections of human germline cells by ancient exogenous retroviruses [6,7]. Throughout the long evolution of humans, extensive inactivating mutations have accumulated in the encoded sequences of HERVs, resulting in the inability of most HERV sequences to encode functional proteins due to replication defects [8,9]. However, a small number of HERVs retain some degree of transcriptional activity. Normally, they operate during embryonic development but remain silent in mature somatic cells, which is tightly controlled by epigenetic mechanisms [10,11,12,13]. Under epigenetic dysregulation, HERVs may be reactivated and trigger inflammatory responses, neurodegeneration, and even malignancies [13,14,15,16]. The exact relationship between the activation of HERVs and the initiation and progression of melanoma remains unclear, but this is a promising area of research. Therapeutic agents targeting HERVs may provide potential benefits for melanoma patients. In this review, we summarize the potential role of HERVs in the pathophysiology of melanoma and explore the possibility of HERVs as therapeutic targets.

## 2. The Epidemiology, Pathogenesis, and Current Treatment of Melanoma

The incidence and mortality rates of melanoma vary greatly worldwide, contingent upon the availability of early detection and timely intervention. The 2022 global cancer statistics show that almost half of all melanoma cases worldwide occur in Europe (44.1%), followed by North America (34.0%) and then Asia (7.5%) [17]. The top three melanoma mortality rates were Europe (44.6%), Asia (22.4%), and North America (14.9%) [17]. Lighter complexion is a major risk factor for melanoma. Europeans have lighter skin pigmentation and are more susceptible to the carcinogenic effects of solar radiation [18]. The risk of melanoma generally increases with age, and the 2020 European cancer statistics show that people aged 65 years and over are at high risk of melanoma [19]. Currently, the most commonly used staging system for treatment selection and prognosis assessment is the American Joint Committee on Cancer (AJCC) staging system [20,21]. Higher cancer stages indicate more extensive cancer and generally a worse prognosis. To date, extensive marginal resection of the tumor remains the primary treatment for all stages of melanoma. For patients diagnosed in an early stage, surgery is considered an effective cure with a good prognosis [22,23]. In contrast, for locally advanced or metastatic melanoma, surgical treatment alone will not be curative, so necessary adjuvant therapies are required after surgery [23,24].

The initiation and progression of melanoma are influenced by multiple factors, including environmental and genetic factors. Ultraviolet (UV) radiation exposure is the most important environmental risk factor for melanoma [25,26]. UV radiation can cause somatic mutations. It has been confirmed that UV-induced mutations dominate melanoma-related mutations [27]. Hereditary melanoma is relatively rare, accounting for about 10% of all cases [28]. The vast majority of melanomas are caused by somatic mutations [29]. At present, four genomic subtypes have been identified: the BRAF mutant, RAS mutant, NF1 mutant, and triple wild-type [29]. The most critical signaling pathways involved are the mitogen-activated protein kinase (MAPK) pathway and the phosphoinositol-3-kinase (PI3K)/AKT pathway [30,31,32]. The identification of genomic subtypes offers the possibility of targeted therapy for melanoma. To date, the inhibitors that have been shown to be effective and approved for clinical application are those targeting BRAF-mutated subtypes [33,34,35]. In addition, combination therapy with a BRAF inhibitor plus a MEK (a critical component of the MAPK pathway) inhibitor has been shown to reduce toxic effects and improve overall survival compared with BRAF inhibitors alone [36,37]. However, patients who have received targeted therapy for melanoma often develop resistance and experience cancer recurrence [38]. 

Immunotherapy has a wider application in the treatment of patients with melanoma, not limited to the type of mutation [24,39]. For patients with melanoma requiring adjuvant therapy, whether BRAF wild-type or BRAF mutant, immunotherapy is the preferred adjuvant treatment option [24,39]. The most widely used are immune checkpoint inhibitors, that is, the combination or monotherapy of anti-programmed cell death protein 1 (PD-1) and anti-cytotoxic T lymphocyte antigen 4 (CTLA-4) antibodies [24,39,40]. Immune checkpoint inhibitors can relieve the suppression of immune function by blocking the binding of immune checkpoints to their ligands, thereby reactivating immune cells to exert anti-tumor effects [40].

## 3. The Classification, Structure, and Function of HERVs

In 1950, Barbara McClintock identified loci in maize that could move between chromosomes [41]. This discovery facilitated the examination of transposable elements. Subsequently, it was found that 45% of the human genome consists of transposable elements [42]. Endogenous retroviruses (ERVs) are categorized as long terminal repeat (LTR) retrotransposons within class I transposable elements, originating from exogenous retroviruses that have infected the germ line over evolutionary time [43]. 

**Figure 1:**
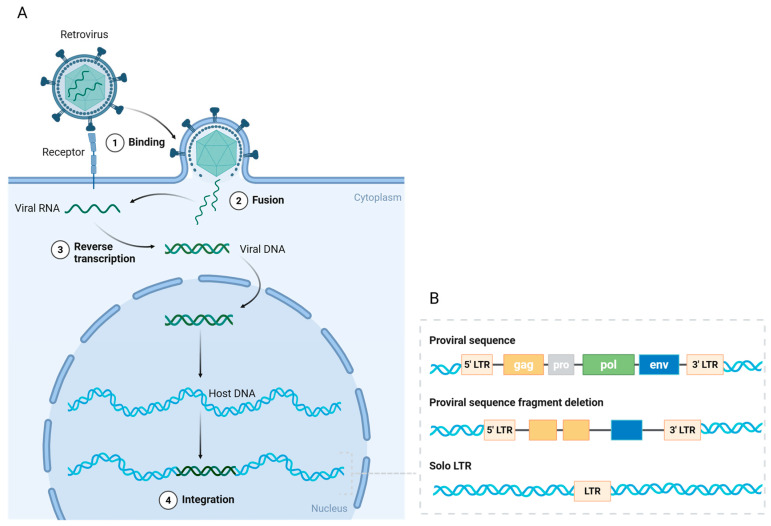
The endogenous transformation and structure of HERVs. (**A**) Retroviruses bind to receptors on germline cells and subsequently fuse with the cell membrane, releasing viral RNA. In the cytoplasm, the viral RNA is reverse-transcribed into viral DNA with the assistance of reverse transcriptase. The viral DNA is transported into the nucleus and integrates into the host DNA, forming a provirus. (**B**) The complete proviral sequence contains open reading frames (ORFs) flanked by long terminal repeats (LTRs). The ORFs include *gag* (group antigen), *pro* (protease), *pol* (polymerase), and *env* (envelope protein) genes. Most ORFs have been deleted or mutated during evolution, with only some retaining their coding sequences. The vast majority of HERVs exist as solitary LTRs. Created in https://BioRender.com.

Human endogenous retroviruses (HERVs) were first described in 1981, and human genome sequencing revealed that they make up 8% of the human genome [6,44,45]. Millions of years ago, exogenous retroviruses invaded primate germline cells, integrated DNA into the genome of host cells, and transmitted vertically to offspring (Figure 1A) [7,46]. Proviruses, which are completely integrated DNAs, remain permanently linked to the infected cell and its progeny unless stochastic mutational events lead to their deletion. During cell division, the integrated provirus replicates with the host cell DNA, permanently becoming a part of the host genome [7,46]. HERVs are divided into three major classes based on sequence similarities in their polymerase genes: Class I (gammaretrovirus-like), Class II (betaretrovirus-like), and Class III (spumaviruses-like) [47,48]. These classes are subdivided into a minimum of 31 families, categorized according to their transfer RNA (tRNA) primer binding sites [47,48]. The letters at the conclusion of each HERV (HERV-K, HERV-H, HERV-W, etc.) denote the tRNA specificity of the primer binding site.

HERVs possess an identical genomic architecture to exogenous retroviruses. Proviral sequences comprise the *gag* (group antigen), *pro* (protease), *pol* (polymerase), and *env* (envelope protein) genes, together with long terminal repeats (LTRs) positioned at both termini (Figure 1B) [6,49]. These four genes encode structural or functional proteins critical for replication-competent retroviruses, while LTRs encompass transcription regulatory regions. Retrovirus replication entails the reverse transcription of the RNA genome from viral particles into DNA, which subsequently integrates into the host cell DNA (Figure 1A). However, inside the human genome, the majority of open reading frames (ORFs) of genes expressed in HERVs have been deleted or mutated throughout evolution, with only a limited number of HERVs preserving all the structural characteristics essential for viral replication (Figure 1B) [8,9]. The timing of provirus integration into the human genome during evolution correlates with the extent of its structural preservation [7,9]. HERV-K is the youngest family and contains proviruses with seemingly intact ORFs [7,9].

The majority of HERVs are present as solitary LTRs as a result of homologous recombination (Figure 1B) [50]. These HERV LTRs retain the potential to regulate gene transcription and contain promoters, enhancers, transcription factor binding sites, splice sites, and polyadenylation signals [51]. It has been proposed that HERVs may function actively without transcription, serving as promoters or enhancers of gene expression [52,53,54]. The repression of HERV expression is also regulated by epigenetic mechanisms, mainly including DNA methylation and histone modifications [13,55]. The evolutionary age of LTRs determines the type of epigenetic control in which they reside [55]. In this respect, humans differ from other mammals, and in particular from mice, which possess a complete sequence of replication-competent ERVs [56,57]. Therefore, the mouse serves as an exemplary animal model for the investigation of ERVs.

Evidence indicates that HERVs contribute to early embryonic development [13]. In embryonic stem cells, the LTRs of HERV-H serve as enhancers and exhibit transcriptional activity during human preimplantation development [52,58]. Syncytins, predominantly expressed in the placenta, facilitate trophoblast fusion and suppress the maternal immunological response against the developing embryo [10,11]. In humans, syncytins include syncytin-1 and syncytin-2, which are encoded by the *env* gene of HERV-W and HERV-FRD, respectively [10,11]. Various studies have demonstrated that HERVs play a role in intricate neuronal development [12,59,60]. Moreover, HERVs govern human embryonic development at the epigenetic level [12,61]. HERVs are generally unable to express functional proteins in mature somatic cells. Infection, radiation, or other external stimuli can aberrantly activate HERVs [13,62,63,64,65,66]. The host immune system can identify specific DNA, RNA, or other intermediates produced by activated HERVs, thus eliciting an inflammatory response [67]. The aberrant expression of HERVs correlates with numerous illnesses, including cancer, neurodegeneration, and autoimmune diseases [14,67,68]. While no definitive evidence suggests that HERVs are carcinogenic, numerous studies have demonstrated that HERVs and their products participate in diverse processes of cancer initiation and progression [68].

## 4. Human Endogenous Retrovirus K

Late-integrated HERVs into the human genome exhibit more intact open reading frames and fewer mutated regulatory sequences compared to ancient elements [7,9]. HERV-K is the latest and most conserved family, capable of producing virus-like particles, particularly its subgroup HML-2. The HERV-K family is classified within the betaretrovirus genus. It was initially identified due to its significant homology with the Mouse Mammary Tumor Virus (MMTV) [69]. Currently, the HERV-K clade has ten groups (designated HML1–10), which are sorted based on the sequence of the reverse transcriptase gene [70]. HERV-K is the only HERV identified as possessing an insertional polymorphism. Insertional polymorphism refers to differences in the presence or absence of HERV sequences in the genomes of different individuals [71]. Not all individuals possess retroviruses at particular genetic loci. Due to recent integration, maintained functionality, and variations in population distribution, insertionally polymorphic HERVs are more significant than ancient and ubiquitous HERVs in pathogenic studies [72,73,74]. The study of the human genome indicated that the insertion of HERV-K (HML-2) varies a lot among individuals [72,73,74]. HERV-K (HML-2) shows a complex and sample-specific transcription pattern in melanoma, and some loci may be used as potential biomarkers or therapeutic targets [75].

Similar to all HERVs, HERV-K insertions have amassed a significant number of deletions and mutations. Nevertheless, HERV-K (HML-2) has preserved complete ORFs that could encode functional proteins [73,76]. Research showed that HERV-K (HML-2) is extensively expressed in healthy cells and tissues, particularly elevated levels in the brain, thyroid, and reproductive tissues [77,78]. It is classified as type I and II viruses based on the presence or absence of a 292-bp deletion in the *pol-env* gene area, with type I producing Np9 and type II producing Rec, both of which are transcribed by the *env* gene [79,80]. Np9 and Rec have been intensively studied for their potential involvement in human cancer [81,82,83]. *HML-2-env* has also been proposed as a possible carcinogenic stimulus [84].

## 5. HERVs Trigger Immune Responses

HERVs in the thymus are epigenetically silenced, and their low expression may facilitate the establishment of central tolerance, resulting in the elimination of some T cells while preserving certain HERV-specific T cells [85,86]. HERVs have also been found in other healthy tissues besides the thymus, and the organism demonstrates tolerance to HERVs to prevent autoimmune responses [85,86,87]. However, the aberrant activation of HERVs during inflammation, infection, or malignancy can disrupt tolerance and initiate adaptive immune responses. The murine leukemia virus (MLV) model in mice has shown that infectious MLV can induce anti-Env antibodies even in the presence of central tolerance, indicating that peripheral immune activation is dominant [88,89].

**Figure 2:**
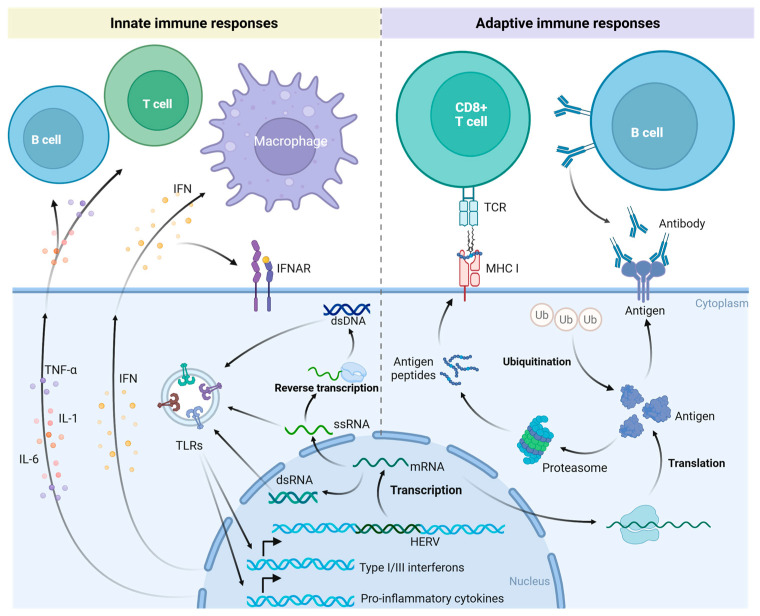
Innate and adaptive immune responses triggered by activation of HERVs. Reactivated HERVs produce dsRNA, ssRNA, and dsDNA, which are recognized by pattern recognition receptors (PRRs) of the innate immune system, such as Toll-like receptors (TLRs). This recognition induces type I and III interferon (IFN) responses and the expression of pro-inflammatory cytokines. These cytokines act on immune cells, initiating innate immune responses. Reactivated HERVs also express novel proteins. These new antigens are presented to CD8+ T cells via major histocompatibility complex (MHC) class I molecules, engaging the adaptive immune response. Some of these antigens may also be directly displayed on the cell surface, where they can be recognized by antibodies secreted by B cells. Created in https://BioRender.com.

Under normal physiological conditions, HERV expression is inhibited. However, HERVs may be reactivated in the presence of illnesses such as cancer. Upon activation, HERVs generate nucleic acid products, including single-stranded RNA (ssRNA), double-stranded RNA (dsRNA), and double-stranded DNA (dsDNA), which stimulate the pattern recognition receptors (PRRs) of the innate immune system, initiate type I and type III interferon (IFN) responses, and provoke an antiviral state (Figure 2) [15,90,91,92,93]. This results in enhanced antigen presentation, the recruitment of immune cells, and the activation of the innate immune response [94]. This process is referred to as “viral mimicry” [92,94]. Upon reactivation under particular conditions, HERVs emulate the molecular characteristics of exogenous retroviruses and trigger the host’s innate immune response. Viral proteins produced by activated HERVs can also elicit adaptive immune responses through B cell and T cell epitopes (Figure 2) [93,94,95,96]. Antibodies against HERV proteins can be detected in a variety of diseases using Western blot, ELISA, immunofluorescence, and other techniques, indicating a wide range of HERV immunogenicity [94,95,97,98,99,100,101,102,103]. The Env and Gag proteins of HERVs are presented by major histocompatibility complex (MHC) class I molecules to activate tumor-specific CD8+ T cells (Figure 2). The HERV Env protein is expressed on the surface of tumor cells and triggers an antibody response (Figure 2). In cancer patients, antibody responses are predominantly observed for the Gag, Env, Rec, and Np9 proteins of HERV-K (HML-2) [104].

The immunogenicity of HERVs is determined by both their residual viral characteristics and the dynamic changes in the host microenvironment. The function of HERV-reactive antibodies in disease is intricate, potentially serving a protective role or contributing to the pathological process. In cancer patients, HERV-reactive antibodies may exhibit multiple effects. When the HERV-K (HML-2) Env glycoprotein is abnormally expressed on the surface of tumor cells, its specific antibody can inhibit the growth and metastasis of tumor cells and induce the apoptosis of tumor cells [105]. HERV-reactive antibodies exhibit synergistic anticancer activity when combined with immune checkpoint inhibitors [106]. The antibody level of HERV-K (HML-2) is associated with the prognosis of cancer patients, and the higher the anti-HERV-K (HML-2) antibody level, the worse the prognosis [107,108,109]. Some HERV-reactive antibodies may also have protumorigenic effects. The Env glycoproteins of HERV-K (HML-2) and HERV-H have been shown to be involved in signal transduction related to tumor initiation and progression, promoting tumor invasion [84,110,111,112]. The multiple roles of HERVs in cancer provide novel insights for treatment.

## 6. The Association of HERV Expression and Melanoma

The search for a link between melanoma and virus-like particles began as early as the 1970s [113,114,115,116]. In 2002, Schiavetti et al. identified a gene with homology to HERV-K in human melanoma cells and designated it HERV-K-MEL [117]. There was a very short open reading frame in the *env* region of HERV-K-MEL, encoding antigenic peptides, which are presented by human leukocyte antigen-A2 (HLA-A2) molecules to CD8+ T cells to generate cytotoxic T lymphocytes (CTLs) [117]. In 2003, Muster et al. showed that human melanoma cells are capable of producing retrovirus-like particles [118]. These particles have reverse transcriptase activity, contain sequences similar to HERV-K, and include mature versions of Gag and Env proteins [118]. Subsequent studies have indicated that HERV-K is active in melanoma tissues and melanoma cell lines, producing nucleic acid products and proteins, and triggering immune responses [98,119,120]. The expression of HERV-K in melanoma may result from the demethylation of CpG sites in the 5′ LTR and enhanced transcriptional activity of the promoter [121,122]. Regarding the association between melanoma and HERVs, most studies focused on HERV-K, and a few studies involved HERV-H. Researchers have conducted a series of explorations on the mechanism of carcinogenesis, the identification of immunological targets, and the application of antiretroviral drugs.

### 6.1. Activation of HERVs

Previously, we discussed the risk factors for melanoma. UV radiation is the most important external risk factor. Research indicates that UV radiation, particularly UVB, enhances HERV-K expression in melanoma cells, resulting in the transcriptional activation of *pol* genes, elevated expression of Env proteins, and the generation of virus-like particles [63]. These findings suggested that HERV-K may play a role in UV-related melanoma pathogenesis. The activation of HERV-K induced by UV radiation may be achieved by affecting epigenetic regulation, which leads to abnormal activation of signal transduction pathways in innate antiviral immunity [64]. 

Aging is also an important risk factor for melanoma. With the passage of time, cells, tissues, organs, and the whole organism undergo slow degenerative changes. Cellular senescence establishes the foundation for organismal aging [123]. Liu et al. found that HERV-K (HML-2) expression was significantly upregulated in senescent cells, accompanied by epigenetic derepression [124]. Activation of HERV-K (HML-2) can also produce retrovirus-like particles and induce senescence in young cells [124]. This process can be blocked by neutralizing antibodies or reverse transcriptase inhibitors [124]. This study suggested that reactivation of HERVs is a hallmark and driving force of aging. 

Cancer-specific transcription factors have been demonstrated to activate HERVs. Microphthalmia-associated transcription factor (MITF) is a lineage-specific transcription factor for melanocytes and melanoma [125]. It regulates pigment synthesis, cell cycle, and survival-related genes, and iit s regarded as an oncogene in melanoma [125]. MITF isoform M (MITF-M) drives HERV-K specific activation in melanocytes and melanomas by directly binding to regulatory sequences in HERV-K LTR [126].

Poor dietary habits, exogenous viruses, and ionizing radiation are also possible activators of HERVs [90,127,128]. The activation of HERVs is intricately linked to epigenetic dysregulation, regardless of whether it is induced by external or internal factors.

### 6.2. Oncogenic Role of HERV-Derived Proteins

**Figure 3:**
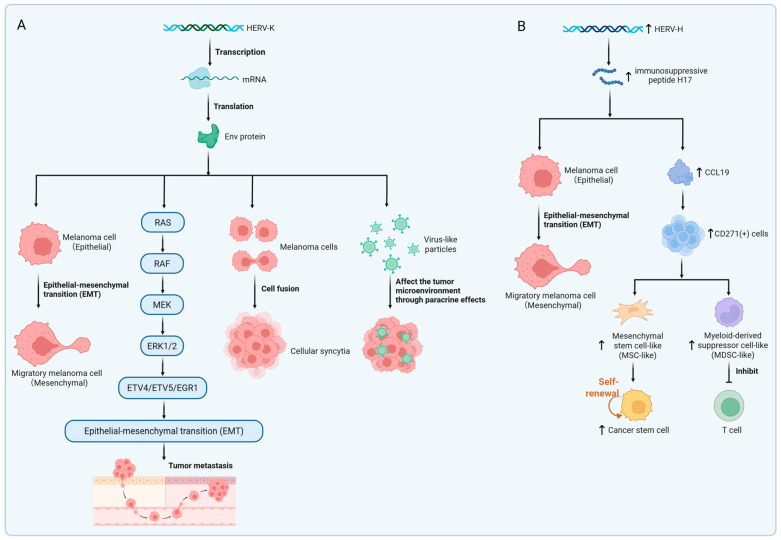
HERV-mediated oncogenic mechanisms. (**A**) Transcriptional expression of HERV-K Env protein. HERV-K Env promotes epithelial–mesenchymal transition (EMT) and tumor metastasis by specifically activating the ERK1/2 signaling pathway. Additionally, HERV-K Env facilitates tumor cell fusion, resulting in syncytia formation. HERV-K-derived virus-like particles influence the tumor microenvironment via paracrine signaling. (**B**) Oncogenic effects of upregulated HERV-H expression. The immunosuppressive peptide H17, derived from the HERV-H transmembrane protein, induces EMT and enhances tumor invasiveness. HERV-H induces the secretion of CCL19, recruiting CD271+ immunoregulatory cells. MSC-like cells promote cancer stem cell expansion. MDSC-like cells suppress T cell activity. Created in https://BioRender.com.

HERV-K (HML-2) Rec protein was positive in 14% of melanoma tissues and was not detected in normal tissues [120]. Singh et al. showed that in proliferative melanoma, a positive feedback loop of Melanocyte Inducing Transcription Factor (MITF)-Rec is formed to promote mutual expression [129]. The regulatory loop formed by MITF and Rec maintains the proliferation of melanoma cells by inhibiting epithelial–mesenchymal transition (EMT) but inhibits their transition to an aggressive phenotype (Figure 3A) [129]. EMT refers to the process in which epithelial cells lose their typical epithelial characteristics and gain mesenchymal cell properties. Cancer cells acquire migration and invasion abilities through EMT [130]. Melanoma tissues and cell lines specifically express HERV-K Env protein, but melanocytes express it minimally [97,118,120]. HERV-K Env protein can induce malignant transformation of melanoma cells, reduce their immunogenicity, and facilitate tumor immune escape [63,131,132]. HERV-K Env protein mediates fusion between melanoma cells to form cellular syncytia, leading to genetic variation and promoting tumor progression (Figure 3A) [133]. Env protein activates the ERK1/2 signaling pathway, induces EMT, and enhances cell invasion (Figure 3A) [84]. HERV-K (HML-2) can generate non-infectious virus-like particles in melanoma cells, which may affect the tumor microenvironment through paracrine effects (Figure 3A) [75].

HERV-H may stimulate antitumor immunity or facilitate immunosuppression, depending on factors such as protein isoforms and the microenvironmental context [134]. Hs294T is a human cutaneous melanoma cell line originally derived from metastatic melanoma tissue of a patient [135]. HERV-H expression was low when Hs294T was not stimulated, but after treatment with TGF-β, an EMT inducer, HERV-H mRNA expression was significantly increased [110]. Upon stimulation with the H17 peptide, a 17-aminoacid peptide derived from the immunosuppressive domain of HERV-H, Hs294T cells exhibited enhanced invasive ability and upregulation of mesenchymal markers (Figure 3B) [110]. As a low-HERV-H-expression model, Hs294T revealed a cascade mechanism by which HERV-H induces CCL19 secretion via the H17 peptide, subsequently recruiting and expanding CD271+ immunosuppressive cells (Figure 3B) [110]. HERV–H LTR-associating 2 (HHLA2), a type I transmembrane protein exclusively found in primates, exhibits dual immunomodulatory functions [136]. HHLA2 is widely expressed in melanoma but scarcely detected in corresponding normal tissues [137]. Its expression pattern is largely mutually exclusive with PD-L1, suggesting its potential as a novel therapeutic target for immunotherapy in patients resistant to PD-1/PD-L1 inhibitors [134,138,139].

### 6.3. HERVs as a Biomarker of Melanoma

Human melanoma and metastases express the HERV-K protein, whereas melanocytes and normal lymph nodes hardly express it [118]. Humer et al. detected HERV-K-specific antibodies in the serum of patients with melanoma [98]. A statistically significant difference existed in the seroprevalence of this antibody between melanoma patients and healthy people [98]. Elevated HERV-K antibody titers in the serum of patients with melanoma are associated with a poor prognosis [140]. The specific expression of HERV-K proteins in melanoma cells and high antibody titers in patient sera suggest that they may be ideal targets for the diagnosis of melanoma. Although the expression of the HERV-K protein is associated with the malignant phenotype of melanoma, it is not expressed in all melanomas, suggesting that the HERV-K protein may serve as a copredictor rather than an independent oncogenic factor [120]. In addition, there was no significant difference in HERV-K protein expression between patients with different stages of melanoma [98]. In 2013, Schmitt et al. identified a melanoma-specific locus, ERVK-6, which is only transcribed in melanoma samples and may serve as a biomarker for melanoma [75]. In 2022, Bendall et al. characterized the specific HERV expression landscape through locus-specific HERV analysis and demonstrated that differential HERV expression can distinguish metastatic from nonmetastatic uveal melanoma [141]. Locus-specific HERV analysis reveals the potential role of these “genomic fossils” in human health and disease by focusing on specific viral insertion sites. Combined with high-throughput sequencing and gene editing technology, this field is becoming a hot spot in the intersection of genomics and virology.

### 6.4. HERVs May Be Associated with the Stem Cell Phenotype of Melanoma Cells

The cancer stem cell phenotype refers to a small subset of cells within a tumor that exhibit stem cell-like properties. These properties enable them to play critical roles in tumor initiation, progression, metastasis, drug resistance, and recurrence [142,143]. Such cells are referred to as cancer stem cells (CSCs). In melanoma, CD133-positive (CD133+) cells are considered a subpopulation with stem cell properties [144]. In a stem cell medium, melanoma cells underwent a phenotypic switch to form non-adherent grape-like aggregates, accompanied by an increase in the proportion of CD133+ cells and upregulation of HERV-K expression [145]. RNA interference experiments showed that the inhibition of HERV-K significantly reduced the expansion of CD133+ cells and impaired their stemness characteristics [145]. Non-nucleoside reverse transcriptase inhibitors (NNRTIs) can inhibit HERV-K activity, lead to a significant reduction in the proportion of CD133+ cells, and selectively induce apoptosis of CD133+ cells [145]. These results suggested that HERV-K activation is closely associated with the expansion of the CD133+ melanoma stem cell subpopulation. It promotes tumor progression by maintaining stemness characteristics. Similar experimental ideas also demonstrated that HERV-H activation is involved in the maintenance of the stemness characteristics of melanoma cells [146]. HERV-K is also involved in maintaining the stem cell-like properties of tumor cells in other cancer types. A study by Shah et al. demonstrated that HERV-K supports the maintenance of glioblastoma stem-like cells through mTOR pathway activation, further reinforcing the broader oncogenic role of HERV-K in sustaining cancer stemness across various tumor types [147].

## 7. Therapeutic Implications of HERVs in Melanoma

The exploration of HERVs in cancer therapy involves leveraging both their viral and immunogenic properties, as well as their potential as novel therapeutic targets [68,148]. Here, we discuss possible strategies targeting HERVs for the treatment of melanoma.

### 7.1. Epigenetic Drugs Activate HERVs

**Figure 4:**
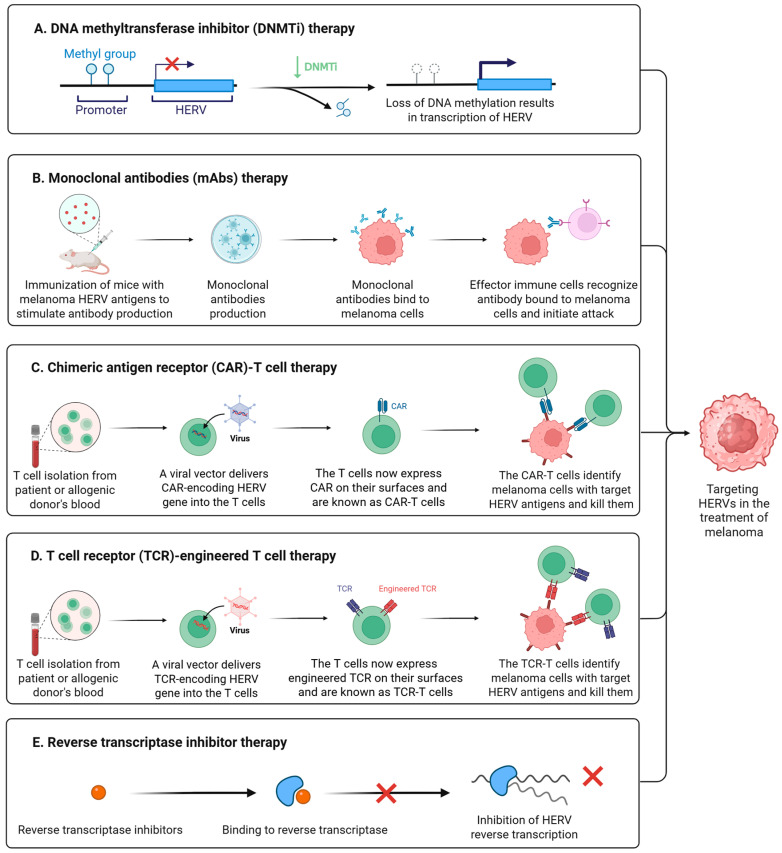
Targeting HERVs in the treatment of melanoma. (**A**) DNA methyltransferase inhibitors (DNMTis) promote transcriptional activation of HERVs through suppression of DNA methylation-mediated epigenetic silencing. (**B**) Monoclonal antibodies (mAbs) generated through HERV antigen immunization specifically bind to melanoma cells, enabling immune effector cells to engage antibody-coated targets and mediate cytotoxic elimination. (**C**) Chimeric antigen receptor (CAR)-T cells specifically target and eliminate melanoma cells expressing HERV surface antigens. (**D**) T cell receptor (TCR)-engineered T cells specifically recognize and eliminate melanoma cells presenting HERV antigens. (**E**) Reverse transcriptase inhibitors bind to reverse transcriptase and inhibit the reverse transcription process of HERVs. Created in https://BioRender.com.

As we mentioned earlier, epigenetic drugs may activate HERVs by inhibiting their epigenetic regulation. Activated HERVs trigger viral mimetic effects and enhance antitumor immunity. The most common epigenetic drugs are DNA methyltransferase inhibitors (DNMTis) and histone deacetylase inhibitors (HDACis). DNMTis stimulate the production of HERV-derived dsRNA in tumor cells, trigger IFN responses through the MAD5 or TLR3 signaling pathways, induce tumor cell apoptosis, and enhance immune infiltration (Figure 4A) [15,91]. Using DNMTis in cancer treatment could raise the levels of CTLA-4 and PD-L1, which may help the cancer respond better to immune checkpoint inhibitor therapy [149]. The combination of epigenetic drugs and immune checkpoint inhibitors may have important clinical implications [150]. In 2021, a study by Zhou et al. revealed that activation of the tumor suppressor protein p53 triggers the reactivation of HERVs, inducing a viral mimicry response and enhancing anti-tumor immune responses [151]. p53 achieves this by binding to HERV promoters and suppressing epigenetic regulators responsible for their silencing, thereby lifting repression on HERVs [151]. For melanoma patients, MDM2 inhibitors, which activate p53, were shown to convert "cold tumors" into "hot tumors," thereby improving the efficacy of immune checkpoint inhibitors [151].

### 7.2. Monoclonal Antibodies Targeting HERVs

Monoclonal antibodies (mAbs) are highly specific antibodies produced by a single B-cell clone [152]. They target a single epitope of a specific antigen, exhibiting high homogeneity and specificity [152]. The HERV-K Env protein is highly expressed in breast cancer and melanoma. In 2012, Wang et al. developed monoclonal antibodies against HERV-K Env [105]. In a breast cancer xenograft model using immunodeficient mice, the 6H5 antibody treatment group showed a significant reduction in tumor volume [105]. Monoclonal HERV-K Env antibodies demonstrate therapeutic potential in breast cancer by inhibiting proliferation, inducing apoptosis, and regulating key signaling pathways [105]. This study highlights that HERV-targeting monoclonal antibodies may emerge as a promising novel direction for immunotherapy in breast cancer and melanoma (Figure 4B).

### 7.3. CAR-T/TCR-T Therapy Targeting HERVs

A chimeric antigen receptor (CAR) is an artificially synthesized receptor protein created through genetic engineering, primarily used to modify T cells to enhance their ability to target and eliminate specific antigens [153]. CAR-T therapy is an immunotherapy that employs genetically engineered CAR-T cells to treat cancer (Figure 4C) [153]. In 2015, Krishnamurthy et al. explored the feasibility of targeting the HERV-K Env protein using CAR-T cells for the treatment of advanced melanoma [154]. In vitro experiments demonstrated that CAR-T cells specifically killed HERV-K Env protein-positive melanoma cell lines and recognized shed HERV-K Env antigens from tumor cells [154]. Mouse models showed that CAR-T treatment significantly inhibited the growth and metastasis of melanoma while demonstrating good safety profiles [154]. In the same year, Zhou et al. reported similar anti-tumor effects of CAR-T cells targeting HERV-K Env protein in breast cancer research [155]. The significant anti-tumor activity of HERV-K-specific CAR-T cells observed in preclinical studies provides a novel direction for the immunotherapy of advanced melanoma and other solid tumors.

TCR-T therapy is a form of adoptive cell therapy that involves genetically engineering a patient’s T cells to express specific T cell receptors (TCRs), enabling them to recognize tumor internal antigens presented by HLA molecules (Figure 4D) [156]. Unlike CAR-T therapy, TCR-T can target intracellular protein fragments, expanding the scope of therapeutic targets. In 2022, Bonaventura et al. screened HLA-A2-focused HERV epitopes associated with cytotoxic T lymphocyte (CTL) responses [157]. Experiments demonstrated that these epitopes could induce high-avidity CD8+ T cell clones and effectively kill HLA-A2+ tumor cells [157]. In triple-negative breast cancer (TNBC) patients, these HERV epitopes were highly expressed, and epitope-specific T cells were detected in tumor-infiltrating lymphocytes (TILs) [157]. Patients with high expression of these epitopes showed significantly improved survival rates [157]. The identified HERV-derived epitopes are highly immunogenic, capable of inducing high-avidity T cells to kill tumors, and are correlated with improved patient survival [157]. These findings provide novel targets for developing universal cancer vaccines or TCR-T therapies for tumors with low mutational burden, such as breast cancer, melanoma, and others.

Targeting HERVs for immunotherapy remains controversial [158,159]. While elevated HERV expression is observed in numerous tumor cells, it is also constitutively detectable in embryonic stem cells and other normal cells. Whether such therapies may indiscriminately target healthy cells and tissues, potentially compromising reproductive function, has yet to be determined. We argue that novel therapeutic development inevitably faces debate, necessitating rigorous safety evaluations.

### 7.4. Antiretroviral Drugs Inhibit HERV Activation

The genomic structural similarities between HERV and HIV suggest that the antiretroviral drugs used to treat HIV may also inhibit HERV activity. Although antiretroviral drugs have not been approved for the treatment of melanoma, several reverse transcriptase inhibitors have been shown to affect tumor cell growth and differentiation [160]. In 2005, Sciamanna et al. used efavirenz to inhibit the growth of human melanoma xenografts expressing HERV-K [119]. Efavirenz is a representative drug for non-nucleoside reverse transcriptase inhibitors (NNRTIs) (Figure 4E). In papers published in 2017 and 2019, Matteucci et al. demonstrated that in vitro treatment of melanoma cell lines with NNRTIs downregulates the transcriptional activity of HERV-K and HERV-H, reduces the number and volume of spheroid aggregates, and induces apoptosis [145,146]. In 2024, Zanrè et al. demonstrated that lamivudine, doravirine, and cabotegravir all downregulate the expression of HERV-K *pol* and *env* genes in melanoma cells, with cabotegravir exhibiting the most pronounced effect [161]. Lamivudine, doravirine, and cabotegravir are nucleoside reverse transcriptase inhibitors (NRTIs), non-nucleoside reverse transcriptase inhibitors (NNRTIs), and Integrase Strand Transfer Inhibitors (INSTIs), respectively. These drugs exhibit no significant toxicity toward normal epithelial melanocytes but exert specific inhibitory effects on melanoma cells with BRAF and p53 mutations [161]. Shortly afterward, the team published another study that demonstrated that in BRAF-mutated melanoma cell lines that had developed resistance to BRAF inhibitors, the use of cabotegravir or doravirine could suppress HERV-K activity, reverse the BRAF-inhibitor-induced upregulation of HERV-K, and induce apoptosis in drug-resistant tumor cells [162], providing new avenues for developing combination therapies.

## 8. Conclusions and Future Perspectives

In recent decades, the relentless exploration of HERVs has profoundly advanced our understanding of their biological roles. Although no conclusive evidence currently substantiates a causative role of aberrant HERV activation in direct tumorigenesis, accumulating in vitro and in vivo experimental evidence implicates HERVs as active participants in oncogenic initiation and progression. This review focuses on delineating the interplay between HERVs and melanoma pathogenesis. First, we analyze the distinct biological characteristics of melanoma and HERVs. Second, we examine their mechanistic connections through the lens of tumor biomarkers and immunotherapeutic targets. Our synthesis posits that HERV-derived elements are aberrantly expressed in melanoma, correlating with malignant transformation, while melanoma exhibits responsiveness to diverse HERV-targeted therapeutic modalities. The strategic integration of HERV-directed therapies with immune checkpoint inhibitors represents a promising avenue for melanoma treatment.

Regarding HERV-targeted oncology interventions, concerns persist about potential off-target effects due to basal HERV expression in normal tissues. Crucially, identifying tumor-specific HERV isoforms or epitopes is imperative to ensure therapeutic specificity. With accelerating advances in genomics, transcriptomics, and emerging single-cell omics technologies, we anticipate the elucidation of the regulatory mechanisms governing HERV activation in malignant contexts. Novel HERV-centric therapeutic platforms hold immense potential to revolutionize precision oncology in the future.

## Data Availability

No new data were created or analyzed in this study.
